# Effects of coaches' transformational leadership behavior on team cohesion: the mediating role of mental toughness

**DOI:** 10.3389/fpsyg.2025.1692882

**Published:** 2025-11-25

**Authors:** Yifan Peng, Ming Yu Claudia Wong

**Affiliations:** Department of Health and Physical Education, The Education University of Hong Kong, Tai Po, Hong Kong SAR, China

**Keywords:** coach behavior, transformational leadership, team cohesion, mental toughness, sports psychology, college athletes

## Abstract

The leadership behavior of coaches has always been the focus of research in the field of sports, and the effect of coache' leadership behavior on team cohesion is even more of a hot research issue. Previous studies have shown that coach leadership behaviors influence individual athlete' participation in sports, thereby affecting team cohesion. Compared with traditional leadership styles, transformational leadership behavior focuses on the interaction between coaches and athletes, so more and more scholars have begun introducing transformational leadership behavior into research related to sports coaching. Additionally, mental toughness as a psychological ability plays an important role in athletes facing challenges and adversity, and is also a factor worth studying. However, current research has not yet fully explored the relationship between coache' transformational leadership behavior, team cohesion, and mental toughness, so this may be an area worthy of further research to fill some gaps in existing research. This study was a cross-sectional study, 123 high-level athletes in colleges and universities of China completed the Transformational Leadership Questionnaire, Group Cohesion Questionnaire, and Sports Mental Toughness Questionnaire. SPSS statistical software was used to conduct descriptive, Pearson correlation, and mediation analysis of the collected questionnaire data, to verify whether mental toughness plays a mediating role between coache' transformational leadership behavior and team cohesion. The results showed that coaches' transformational leadership behavior perceived by college athletes were associated with the team cohesion and the mental toughness of athletes, mental toughness partially mediated the relationship between transformational leadership and team cohesion, with a mediating effect of 16.34%. The results provided a basis for improving the coach's leadership behavior, coaching philosophy, and promoting team cohesion.

## 1 Introduction

Team cohesion is an important factor affecting team success, and it is also the source of strength that brings teams together to achieve common goals (Carron et al., 2002). In sports competitions, teams strive to achieve the best performance to achieve better competition results. Team cohesion has also received widespread attention due to its important role in sports performance. Moreover, the coach is a pivotal figure within a sports team, acting as an organizer and a guide. Many scholars have confirmed that coach leadership behavior is a key factor affecting team cohesion (Westre and Weiss, 1991; You, 2014; Callow et al., 2009; Vincer and Loughead, 2010; Cronin et al., 2015; Smith et al., 2013). In leadership behavior, research indicates that coaches' leadership behaviors significantly impact team cohesion by fostering athletes' sense of belonging and dependence on the team, enhancing their technical skills, and addressing their individual needs (Salcinovic et al., 2022). Conversely, coaches' authoritarian leadership behavior can cause athletes to lose their voice, their personal wishes to be ignored, and they can only passively accept various decisions, which may reduce investment and undermine team cohesion. Salcinovic et al. (2022). Leadership behavior may affect team cohesion by affecting the attractiveness of the team to members and the degree to which personal pursuits match team goals. Therefore, what kind of leadership behavior coaches adopt to promote team cohesion is an issue that needs to be explored.

As a relatively advanced and scientific leadership theory, transformational leadership has been theoretically associated with positive developmental outcomes in sport settings (Kassim and Hassan, 2020). Transformational leadership Theory (Bass, 1985) has proven to be an appropriate guiding theory for research investigating coaching in sport (Arthur et al., 2011). This creates an environment in which members within the team trust and encourage each other, so that the team can reasonably divide the work, each team member performs their duties, and more unitedly complete the goals pursued by the team (Robbins et al., 2001). Chan and Mak (2014) suggest that transformational leadership has a positive impact on organizational development by increasing the emotional and normative commitment of followers. In contrast, traditional leadership styles, such as transactional leadership, focus more on setting clear goals, providing reward and punishment mechanisms, and monitoring performance, their interactive mode is usually based on the principle of exchange, where athletes complete tasks to receive rewards or avoid punishment (Doherty and Danylchuk, 1996); transformational leadership emphasizes the interaction between coaches and athletes, focusing on establishing an inspiring vision, providing support and challenges for athletes, and deeply stimulating athletes' intrinsic motivation and long-term development (Bass, 1985; Charbonneau et al., 2001). Concurrently, mental toughness is increasingly recognized as a vital psychological trait that underpins athletic success (Gucciardi et al., 2014). Mental toughness is a widely discussed concept in the field of sports psychology. Athletes, coaches and sports psychologists have always believed that mental toughness is one of the most important psychological characteristics related to sports success. A large number of studies have been conducted to investigate the role of mental toughness in sports success, but there is a lack of clarity and consensus on its definition (Liew et al., 2019). This research tends to define mental toughness was defined as the psychological qualities enabling athletes to remain confident, focused, and motivated under stress, and is a key determinant of an athlete's ability to perform well in high-pressure situations (Gucciardi et al., 2009).

In the cultural context of the integration of sports and education in China, college athletes need to cope with the dual pressure of academic and training simultaneously (Hu et al., 2016). They have to complete university courses while ensuring several hours of high-intensity training every day. At the same time, there is often a high demand for competitive level, and athletes from high-level sports teams in some universities have reached a considerable level of competitiveness, achieving excellent results in national and even international competitions. But most university coaches not only undertake coaching work, but also need to give lectures and take on teaching duties, which can easily lead to energy dispersion and difficulty in balancing all responsibilities (Wang et al., 2022; Xu et al., 2025). Due to the requirement for competition results, most coaches often adopt authoritarian leadership behavior, with completing tasks as the core, thus ignoring the athletes' own feelings, which may lead to the decline of team cohesion, or even the psychological problems of athletes, such as anxiety (Jin et al., 2022; Hagerty and Felizzi, 2023). Therefore, the psychological status of Chinese college athletes and the leadership behavior of coaches are important points worth paying attention to.

Based on previous research, no studies have fully explored the relationship between transformational leadership behavior, team cohesion and mental toughness. Therefore, studying the relationship between coaches' transformational leadership behavior, team cohesion and mental toughness, and verifying whether mental toughness can mediate the impact of transformational leadership behavior on team cohesion is a research gap worthy of study. The theoretical significance aims to better explain the relationship between transformational leadership behavior, team cohesion and mental toughness, enrich the research on coaching behavior, team performance and athlete psychology, and the practical significance can provide more useful methods for coach training, improving team cohesion, and enhancing athletes' psychological quality. Therefore, this study has certain theoretical and practical significance.

## 2 Theoretical background and hypothesis development

### 2.1 The relationship between transformational leadership, team cohesion, and mental toughness

The transformational leadership of coaches has been extensively studied in the field of sports, coaches who exhibit transformational leadership behaviors have been shown to be positively associated with various aspects of the team. Many scholars found that transformational leadership was positively associated with team cohesion, coaches who communicate a compelling vision, provide individualized support, and stimulate intellectual growth among athletes enhance the sense of unity and togetherness within the team (Callow et al., 2009; Vincer and Loughead, 2010; Smith et al., 2013; Cronin et al., 2015; Altahayneh and Qatami, 2019), these behaviors map onto the components of transformational leadership, idealized influence/charisma, inspirational motivation, intellectual stimulation, and individualized consideration. Coaches who exhibit these behaviors create an environment where team members feel valued and motivated to work toward common goals, thereby strengthening the bonds within the team. Later research has also shown that coaches who exhibit transformational leadership behaviors can significantly enhance team cohesion (Smith et al., 2013).

In addition, coaches who display behaviors aligned with transformational leadership have been linked to the mental toughness of athletes. Research conducted by Finch (2022) showed that coaching based on the four dimensions of transformational leadership significantly improved athlete' mental toughness and emotional control. This approach enhanced athlete' ability to develop greater resilience and self-confidence. Similarly, Murray et al. (2021) found that transformational coaching was positively associated with mental toughness in adolescent soccer players. The ability of coaches to inspire, motivate, and provide individualized support played a crucial role in enhancing the mental toughness of athletes.

Although empirical research on the relationship between team cohesion and mental toughness is relatively limited compared to the other two relationships, existing studies also suggest a positive link between them. For instance, Ye (2014) found that individuals with higher social support, which can be provided by a cohesive team, tend to have enhanced mental toughness. Cohesion within a team can be positively affected by the collective mental toughness of its members. Additionally, research has also shown that teams with higher levels of mental toughness exhibit greater resilience and adaptability, which enhances their overall performance (Gu et al., 2022). Gu et al. (2022) also confirmed that team cohesion and its various dimensions have a significant positive effect on athletes' mental toughness. Research by Yildirim et al. (2024) indicated that team cohesion is related to athletes' psychological health, both directly and indirectly through basic psychological needs. A cohesive team provides a supportive environment where athletes feel valued and motivated, which can contribute to their mental toughness.

### 2.2 The mediating role of mental toughness

Existing research shows that many factors mediate the relationship between transformational leadership and team cohesion. For example, Mach et al. (2022) found that leadership consensus played a moderating role in the relationship between transformational leadership behavior and team cohesion. Leadership consensus refers to the degree of consistency among team members in their perception of leadership behavior. The psychological motivation derived from past successes combines with a highly consistent perception of leadership to create a synergistic effect, making team members more receptive to the collaborative concepts and goals advocated by transformational leadership, thereby promoting cohesion improvement. Oh and Yoo (2023) suggested that social norms can serve as a mediator between transformational leadership and team cohesion. Social norms, which are the common expectations for behavior within a team, can influence how athletes perceive and respond to their leader's actions, thereby affecting team cohesion. Additionally, Wang et al. (2019) found that athletes' role involvement and training - competition satisfaction mediated the relationship between transformational leadership and team cohesion. When athletes are more engaged in their roles and satisfied with their training and competition experiences, they are more likely to feel a sense of unity with the team. Cronin et al. (2015) further demonstrated that transformational leadership is associated with increased task cohesion, with inside sacrifice acting as a mediating factor. Transformational coaches inspire team members to make sacrifices for the greater good of the team, which in turn promotes a more cohesive team environment.

Notably, while the direct relationship between transformational leadership and team cohesion is well-documented, the potential mediating role of mental toughness remains largely unexplored. Mental toughness can enable athletes to remain confident, focused, and motivated under stress, and is a key factor influencing athletes' performance under high pressure (Gucciardi et al., 2009). Especially college athletes need to cope with the dual pressure of academic and sports at the same time, which may weaken team cohesion. Mental toughness helps them keep balance under such pressure, while not neglecting their responsibilities to the team, so it is particularly important to improve their mental toughness. Drawing on the established relationships among transformational leadership, team cohesion, and mental toughness, it is reasonable to hypothesize that mental toughness may mediate the impact of coaches' transformational leadership behavior on team cohesion. This proposed mediation implies that transformational leadership behavior may not only directly enhance team cohesion but also indirectly strengthen it by cultivating mental toughness among team members.

### 2.3 Research hypotheses

Based on the theoretical background and previous research results, the research hypotheses are as follows:

**H1 Coaches' transformational leadership behavior has a positive influence on team cohesion**.
**H2 Coaches' transformational leadership behavior has a positive influence on mental toughness**.
**H3 Mental toughness mediates the relationship between coaches' transformational leadership behavior and team cohesion**.

## 3 Materials and methods

### 3.1 Participants

This study is a cross-sectional experiment to investigate the relationship between college athletes' perceived transformational leadership behavior of coaches and team cohesion and mental toughness. Therefore, the study selected high-level athletes from China as research subjects. In the context of China, high-level athletes are defined as those who hold a national second-level athlete certificate or above, and have received regular training and participated in provincial and national level competitions, and they are also college athletes from colleges and universities at the same time. This study mainly evaluated the perception of athletes, coaches were not investigated.

According to the calculations applied to the multiple regression analysis in the G*Power software, set Effect size *f*^2^ to 0.15 (medium effect), α err prob to 0.05 (significance level), Power (1−β err prob) to 0.95 (95% certainty of finding an effect), 107 samples were needed. To ensure the validity of the study, a total of 130 questionnaires were distributed this time. After excluding 7 missing and invalid questionnaires, 123 valid questionnaires remained, and the effective recovery rate of the questionnaires was 94.62%.

### 3.2 Measures

This study used the Chinese version of mature questionnaires that have been verified to have good reliability and validity. The questionnaire consists of 56 items, including personal basic information (5 items), transformational leadership questionnaire (24 items), group cohesion questionnaire (15 items), and sports mental toughness questionnaire (12 items), using a 5-point Likert scale ranging from 1 (strongly disagree) to 5 (strongly agree). The questionnaire is collected through online questionnaire platforms and face-to-face paper questionnaires. The online questionnaire was distributed to the target network class group, and the paper questionnaire was distributed after the target sports team training to ensure that the questionnaires were completed by target participants, and the questionnaire includes professional personal information items. The 123 participants in this study were all high-level athletes from university sports colleges who had been verified. Their sports specialties are both team sports, including basketball, football, volleyball, etc. Approval for this research was granted by the Institutional Review Board at a university with which one of the authors is affiliated. The informed consent form was provided to participants, who voluntarily chose to fill it out.

#### 3.2.1 Transformational leadership

This study used the revised questionnaire developed by Li (2010) based on the Transformational Leadership Questionnaire (TLQ) developed by Li and Shi (2005), which is more in line with China's national conditions and has better reliability and validity. The questionnaire consists of four dimensions: Morale Modeling, Charisma, Articulate Vision, and Individual Consideration, with six items in each dimension and a total of 24 items. Higher values indicate stronger transformational leadership. The Cronbach's Alpha coefficient for the questionnaire of transformational leadership was 0.968, and the Bartlett's Test was significant (χ^2^ = 2650.611, df = 276, *p* < 0.001).

#### 3.2.2 Team cohesion

The Group Cohesion Questionnaire (GCQ) was used to measure team cohesion in this study. This questionnaire was adapted by Ma (2008) according to the Group Environment Questionnaire (GEQ) developed by Carron et al. (1985), which is in line with the national conditions of China and has been studied by previous researchers with good reliability. The GCQ consists of 15 items and four dimensions: Individual Attractions to the Group–Task (ATG-T), with three items; Individual Attractions to the Group–Social (ATG-S), with four items; Group Integration–Task (GI-T), with four items; and Group Integration–Social (GI-S), with four items. Higher values indicate stronger group cohesion. The Cronbach's Alpha coefficient for the questionnaire of team cohesion was 0.915, and the Bartlett's Test was significant (χ^2^ = 1345.428, df = 105, *p* < 0.001).

#### 3.2.3 Mental toughness

This study used the Sports Mental Toughness Questionnaire (SMTQ) translated by Wang et al. (2014), it developed by Sheard et al. (2009) through interviews with a large number of athletes was chosen for the study on the basis of its refinement the questionnaire and its appropriateness for localization, and its internal consistency, reliability, and validity were relatively good. The SMTQ consists of 12 items and three dimensions: Confidence, Constancy, and Control, with four items in each dimension. Higher scores indicate greater mental toughness. The Cronbach's Alpha coefficient for the questionnaire of mental toughness was 0.742, and the Bartlett's Test was significant (χ^2^ = 486.561, df = 66, *p* < 0.001).

#### 3.2.4 Statistical analysis

This paper uses SPSS 23.0 software for analysis. Descriptive statistical analysis was used to analyze the categorical variables of personal information in terms of frequency (n) and frequency (%). The descriptive analysis of the basic situation of each variable dimension is performed in the format of mean ± standard deviation. Cronbach's coefficient is used to conduct reliability analysis on coaches' transformational leadership behavior, mental toughness, and team cohesion, and KMO is used to conduct validity analysis on coaches' transformational leadership behavior, mental toughness, and team cohesion. The personal information questions were analyzed for differences in coaches' transformational leadership, mental toughness, team cohesion, and each small dimension. Pearson correlation analysis was used to conduct the correlation analysis on coaches' transformational leadership behavior, mental toughness, team cohesion, and each dimension. Process 4.0 was used to conduct mediation effect analysis to study whether mental toughness has a mediating role between coaches' transformational leadership behavior and team cohesion.

## 4 Results

### 4.1 Results of demographic differences analyses

The general characteristics of the participants were 90 (73.2%) men and 33 (26.8%) women; 96 (78%) participants competed in basketball, and 27 (22%) in other team sports; 70 (56.9%) participants had national second-level athlete certificates, 41 (33.3%) participants had national first-level athlete certificates, and 12 (9.8%) participants had national master athlete certificates; 26 (21.1%) participants aged between 18-20 years old, and 67 (54.5%) participants aged between 21–23 years old, and 26 (21.1%) participants aged between 24–26 years old, and 4 (3.3%) participants aged between 27–30 years old; 27 (22%) participants had 3–5 years of training experience, and 30 (24.4%) participants had 6–8 years of training experience, and 44 (35.8%) participants had 9–11 years of training experience, and 18 (14.6%) participants had 12–14 years of training experience, and 4 (3.3%) participants had above 15 years of training experience (see Table 1).

**Table 1:** Demographic information.

**Item**	**Option**	**Frequency**	**Percent**
Gender	Male	90	73.20%
	Female	33	26.80%
Age	18-20 years old	26	21.10%
	21–23 years old	67	54.50%
	24–26 years old	26	21.10%
	27–30 years old	4	3.30%
Sports	Basketball	96	78.00%
	Other team sports	27	22.00%
Training years	3–5 years	27	22.00%
	6–8 years	30	24.40%
	9–11 years	44	35.80%
	12–14 years	18	14.60%
	15 years above	4	3.30%
Athlete Level	National second-level athlete	70	56.90%
	National first-level athlete	41	33.30%
	National master athlete	12	9.80%

After analyzing demographic differences, the results showed that different demographic variables such as gender, age, sports, and training years do not significantly related transformational leadership, team cohesion, mental toughness and all its dimensions. Only athlete levels showed a limited related to some sub-dimensions of transformational leadership. This suggests that coaches' transformational leadership behavior may be more universally applicable across different demographic groups, and mental toughness may serve as a mediating factor in the relationship between transformational leadership and team cohesion regardless of demographic differences.

### 4.2 Results of correlation analyses

The results of correlation analyses indicated that transformational leadership behavior is positively correlated with team cohesion (*r* = 0.499, *p* < 0.01). Transformational leadership behavior also exhibited strong correlations with the dimensions of team cohesion: ATG-T (*r* = 0.873, *p* < 0.01), ATG-S (*r* = 0.902, *p* < 0.01), GI-T (*r* = 0.940, *p* < 0.01), and GI-S (*r* = 0.892, *p* < 0.01). Additionally, transformational leadership behavior is positively correlated with mental toughness (*r* = 0.344, *p* < 0.01), and team cohesion is likewise positively correlated with mental toughness (*r* = 0.380, *p* < 0.01). Therefore, there is a significant positive correlation among transformational leadership behavior, team cohesion, and mental toughness. The results of descriptive statistics and correlations are shown in Table 2.

**Table 2:** Descriptive statistics and correlations.

**Variables**	**M**	**SD**	**1**	**1A**	**1B**	**1C**	**1D**	**2**	**2A**	**2B**	**2C**	**2D**
1. Transformational leadership	4.22	0.7										
A. Morale modeling	4.12	0.78										
B. Charisma	4.33	0.76										
C. Articulate vision	4.36	0.7										
D. Individual consideration	4.06	0.85										
2. Team cohesion	4.14	0.69	0.50^**^	0.87^**^	0.92^**^	0.93^**^	0.91^**^					
A. ATG-T	4.32	0.77	0.87^**^	0.51^**^	0.64^**^	0.61^**^	0.53^**^					
B. ATG-S	4.36	0.77	0.90^**^	0.43^**^	0.49^**^	0.43^**^	0.36^**^					
C. GI-T	4.27	0.84	0.94^**^	0.43^**^	0.51^**^	0.48^**^	0.44^**^					
D. GI-S	3.84	0.79	0.89^**^	0.38^**^	0.41^**^	0.40^**^	0.33^**^					
3. Mental toughness	3.41	0.55	0.34^**^	0.27^**^	0.34^**^	0.32^**^	0.31^**^	0.38^**^	0.35^**^	0.31^**^	0.35^**^	0.37^**^
A. Confidence	3.82	0.69	0.50^**^	0.39^**^	0.48^**^	0.46^**^	0.48^**^	0.41^**^	0.45^**^	0.39^**^	0.45^**^	0.34^**^
B. Constancy	3.88	0.65	0.28^**^	0.20^**^	0.29^**^	0.30^**^	0.24^**^	0.41^**^	0.35^**^	0.34^**^	0.39^**^	0.37^**^
C. Control	2.55	0.94	0.01	0.02	0.02	–0.01	–0.01	0.08	0.02	0	0.01	0.14

ATG-T, Individual Attractions to the Group-Task; ATG-S, Individual Attractions to the Group-Social; GI-T, Group Integration-Task; GI-S, Group Integration-Social.

^*^p < 0.05, ^**^p < 0.01.

### 4.3 Results of mediation analyses

The coach's transformational leadership behavior was used as the predictor variable, team cohesion as the outcome variable, and mental toughness as the mediating variable; linear regression was used to test the mediation effect. Since there were no significant differences between the demographic variables in the difference analysis, no control variables were added. The regression analysis results are shown in Table 3.

**Table 3:** Regression analysis.

**Regression equation (*****N*** **= 123)**		**Overall fit indices**			**Significance of regression coefficients**	
**Dependent variable**	**Independent variables**	***R***	***R2***	***F***	β	***t***
Y	X	0.499	0.249	40.018^**^	2.060^**^	6.186
					0.493^**^	6.326
M	X	0.344	0.118	16.216^**^	2.278^**^	7.993
					0.269^**^	4.027
Y	X	0.546	0.298	25.478^**^	1.376^**^	3.445
					0.412^**^	5.121
	M				0.300^*^	2.91

^*^p < 0.05, ^**^p < 0.01.

#### 4.3.1 Model 1

The first model regressed team cohesion (*Y*) on transformational leadership behavior (*X*). The regression equation was


Y=2.060+0.493X.

The results indicated that transformational leadership had a statistically significant positive correlation with team cohesion (β = 0.493, *t* = 6.326, *p* < 0.01). Specifically, a one-unit increase in transformational leadership behavior was associated with a 0.493-unit increase in team cohesion. The model explained 24.9% of the variance in team cohesion (*R* = 0.499, *R*^2^ = 0.249), and the overall model was statistically significant (*F* = 40.018, *p* < 0.01). Thus, Hypothesis 1 was supported.

#### 4.3.2 Model 2

The second model regressed mental toughness (*M*) on transformational leadership behavior (*X*). The regression equation was


M=2.278+0.269X.

The results showed that transformational leadership had a statistically significant positive correlation with mental toughness (β = 0.269, *t* = 4.027, *p* < 0.01). A one-unit increase in transformational leadership behavior was associated with a 0.269-unit increase in mental toughness. The model explained 11.8% of the variance in mental toughness (*R* = 0.344, *R*^2^ = 0.118), and the overall model was statistically significant (*F* = 16.216, *p* < 0.01). Thus, Hypothesis 2 was supported.

#### 4.3.3 Model 3

The third model regressed team cohesion (*Y*) on both transformational leadership (*X*) and mental toughness (*M*). The regression equation was


Y=1.376+0.412X+0.300M.

The results revealed that both transformational leadership behavior (β = 0.412, *t* = 5.121, *p* < 0.01) and mental toughness (β = 0.300, *t* = 2.910, *p* < 0.01) had a statistically significant positive correlation with team cohesion. A one-unit increase in transformational leadership was associated with a 0.412-unit increase in team cohesion, while a one-unit increase in mental toughness was associated with a 0.300-unit increase in team cohesion. The model explained 29.8% of the variance in team cohesion (*R* = 0.546, *R*^2^ = 0.298), and the overall model was statistically significant (*F* = 25.478, *p* < 0.01).

The findings of the regression analyses provide evidence of the positive relationships between transformational leadership behavior, mental toughness, and team cohesion. Specifically, transformational leadership behavior significantly enhanced team cohesion directly and indirectly through mental toughness. Thus, Hypothesis 3 was supported.

#### 4.3.4 Bootstrap mediation analysis

Utilizing the bias-corrected percentile bootstrap method, 5,000 repeated samples were drawn to test the mediating effect, with a 95% confidence interval (CI) established. As shown in Table 4, the 95% CI for both the direct and indirect effects excludes zero, indicating significant effects and confirming that mental toughness significantly mediates the relationship between transformational leadership behavior and team cohesion.

- **Direct effect:** The direct effect of transformational leadership behavior on team cohesion is 0.4122, which is statistically significant (95% CI: [0.2528, 0.5715]). This shows that transformational leadership behavior exerts a strong impact on team cohesion even after controlling the indirect pathway through mental toughness.
- **Indirect effect:** The indirect effect of transformational leadership behavior on team cohesion through mental toughness is 0.0805, also statistically significant (95% CI: [0.0322, 0.1457]). This suggests that athletes' mental toughness mediates a portion of the influence of transformational leadership behavior on team cohesion.
- **Total effect:** The total effect of transformational leadership behavior on team cohesion is 0.4927 (95% CI: [0.3385, 0.6469]), which indicates a positive overall relationship between the two variables. A total of 83.66% of this effect arises from the direct pathway from transformational leadership behavior to team cohesion, while 16.34% is mediated by mental toughness.

**Table 4:** Mediation effect table.

**Effect type**	**Effect value**	**Boot SE**	**95%CI**	**Effect size**
Direct effect	0.4122	0.0805	[0.2528, 0.5715]	83.66%
Indirect effect	0.0805	0.0288	[0.0322, 0.1457]	16.34%
Total effect	0.4927	0.0779	[0.3385, 0.6469]	

**Figure 1:**
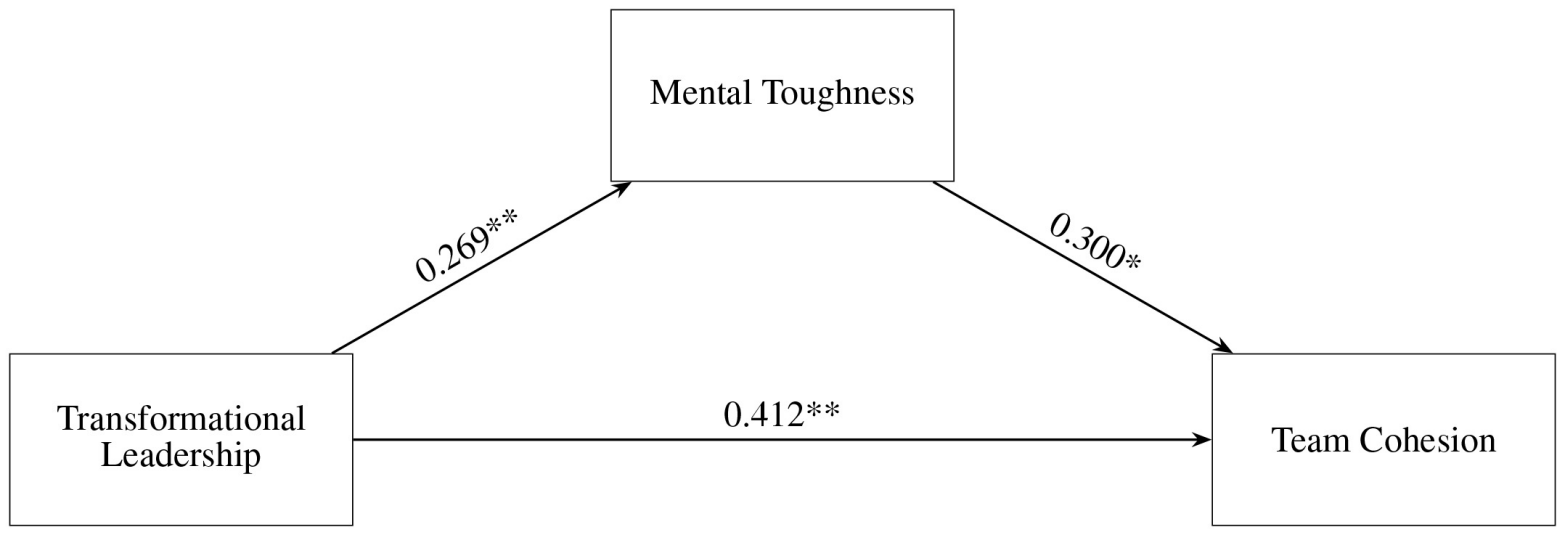
Finalized hypothesized model. ^*^p < 0.05, ^**^p < 0.01.

Thus, Hypothesis 3 was supported again. The mediation effect diagram is shown in Figure 1.

## 5 Discussion

Team leaders play an important role in supporting players' performance and success at the planned level. For this process, coaches must have the qualities to help athletes successfully complete difficult tasks (Kim and Cruz, 2016). In this research, we try to verify the relationship between coache' transformational leadership and team cohesion, and whether mental toughness plays a mediating role in this relationship.

From descriptive statistics, the scores of overall transformational leadership, mental toughness, team cohesion, and internal dimensions are all at a high level (most above 4), and the degree of dispersion is relatively low. Research has confirmed that the benchmark for high transformational leadership is a score of three or more (Bass and Avolio, 1994). It is evident that the team athletes involved in the research appreciate the coach's behavior in improving their abilities and performance. It also shows that the athletes participating in the survey have a strong understanding of coaches' transformational leadership behavior, team cohesion and personal mental toughness.

From the result of demographic differences analyses, in terms of the cognition of transformational leadership, the national master athletes are higher than the national first-level athletes and national second-level athletes. This is consistent with the research results of scholars (Zhou et al., 2005), they considered that athletes with higher athlete levels may have received more systematic training from an early age, and have a higher degree of cognition of coaches' leadership behavior. They can clearly understand the advantages and disadvantages of their technical ability, and show high enthusiasm, consciousness, and self-control ability in training and competition. The national first-level athletes and national second-level athletes may have a short training time and lack of systematic training compared with the national master athletes, and there will be no good consensus when implementing the coach's intention, and the cognition of the coaches' leadership behavior will be weakened. However, the lack of significant differences in team cohesion across various demographic variables suggests that team cohesion may be influenced more by leadership and psychological factors rather than demographic characteristics.

The Pearson correlation results show a significant positive correlation between each dimension of coache' transformational leadership behavior, overall mental toughness, and overall team cohesion. However, the control dimension of mental toughness is not significantly correlated with any dimension of transformational leadership and team cohesion. Perhaps this is because the control dimension of mental toughness is more about an individual's sense of control over the environment and their own behavior, which may be different from the macro level of team cohesion and transformational leadership. According to the theory of mental toughness proposed by Clough et al. (2002), the control dimension is closely related to an individual's internal locus of control. This individual-level psychological attribute may not directly correspond to the team-level construct of team cohesion, which focuses on the unity and cohesion among team members. Therefore, the lack of significant correlation between the control dimension of mental toughness and team cohesion can be explained by the different levels of analysis and theoretical focuses. At the same time, based on the transformational leadership theory developed by Bass (1985), transformational leaders inspire and motivate followers by providing vision, intellectual stimulation, and individualized consideration. This aligns with our finding that coache' transformational leadership behavior has a positive correlation with team cohesion. The results indicated that the more transformational leadership behavior coaches exhibit, the higher the team cohesion. One possible reason for this outcome is that coaches who are perceived to exhibit transformational leadership behavior make team members feel the value of commitment, membership, and teamwork. The changes brought about by coaches have in turn increased athletes' perception of team cohesion. This result is consistent with previous research findings (Callow et al., 2009; Cronin et al., 2015; Smith et al., 2013; Altahayneh and Qatami, 2019), they all suggested that coaches' transformational leadership is closely associated with team cohesion in sports environments. For instance, Callow et al. (2009) and Altahayneh and Qatami (2019) both believed that transformational leadership is effective in not only enhancing athlete motivation and performance, but also in increasing team cohesion. Similarly, Smith et al. (2013) found that transformational leadership behaviors like fostering acceptance of group goals and individual consideration were positively related to task cohesion in sports teams.

Additionally, from the result of mediation analysis, transformational leadership behavior was positively association with team cohesion, transformational leadership behavior was positively association with mental toughness, and both transformational leadership behavior and mental toughness were positively association with team cohesion. The Bootstrap mediation test showed that mental toughness partially mediates the relationship between transformational leadership and team cohesion, with a mediating effect of 16.34% (95% CI [0.0322, 0.1457]). While the direct effect remains dominant (83.66%), the significant mediation effect (16.34%) underscores the value of developing the mental toughness of athletes. The result indicates that coaches can directly enhance team cohesion by improving transformational leadership behaviors and indirectly promote team cohesion by cultivating athlete' mental toughness. Therefore, coaches should focus on improving their leadership abilities while cultivating team members' mental toughness to enhance team cohesion and performance comprehensively.

More specifically, coaches should focus on improving their transformational leadership abilities, including clarifying their expectations for the team, expressing team goals clearly, specifying detailed exercise plans, and encouraging each team member. Secondly, personalized care should be given to each member, paying attention to their personal development and needs, and making clear development plans for each member as much as possible. Finally, they should set a good example by first strictly demanding themselves to achieve the power of role models and enhance trust between teams.

Since mental toughness plays a mediating role between transformational leadership and team cohesion, coaches should also learn to attach importance to cultivating the mental toughness of each team member. Through appropriate stress management training, they can help each member of the team reasonably cope with stress and challenges. Coaches should also follow up on the psychological condition of each team member and provide timely care and feedback, because positive feedback can enhance the team members' self-confidence and ability to withstand pressure. To create a good team atmosphere, each member should be able to encourage and support each other, lend a helping hand when teammates are in difficulty, and increase the mental toughness of each team member, and achieve a win-win situation of team cohesion and individual performance by balancing the needs of the individual and the team.

Specific data shows that coach transformational leadership has a direct effect on team cohesion, accounting for 83.66%. The correlation between the dimensions of articulate vision and group integration–task (GI-T) is the highest (*r* = 0.94), and the correlation between individual consideration and individual attractions to the group–social (ATG-S) is also high (*r* = 0.91). Based on these results, coaches can break down team goals into weekly tasks and display progress on bulletin boards; Establish personal files, conduct one-on-one communication every week, record athletes' technical weaknesses and psychological states, and adjust training accordingly. At the same time, because the control dimension in mental toughness is not significantly related to other variables (*r* = 0.01 − 0.14), athletes can avoid making training plans independently, and can take turns to be the training team leader to practice their sense of control within the team framework. According to the mediation effect of mental toughness, 16.34%, and the dimensions of confidence and constancy are significantly related to team cohesion (*r* = 0.41 − 0.45), coaches can add controllable tasks (such as basketball free throws) after training, and the team applauds after completion; Simulate adverse situations and increase cohesion through methods such as reviewing and collaborating after practice.

The cultivation of coaches' transformational leadership behavior and athletes' mental toughness is not a process that can be achieved overnight, but a process that needs long-term cultivation. It requires continuous input and attention from coaches and team members. Through long-term leadership development and mental toughness cultivation, a solid foundation can be laid for the sustainable development of the team. In the rapid development of today's society, many teams often face a lot of uncertain events and rapidly changing environmental conditions. Coaches should enhance team cohesion and athletes' mental toughness, allowing the team to cope better with uncertainty and maintain the stability and adaptability of the team.

## 6 Conclusion and suggestions

This study explored the impact of transformational leadership behavior of coaches on team cohesion and examined the mediating role of mental toughness. By analyzing the questionnaire data of 123 athletes, the results showed that there was a significant positive correlation between transformational leadership behavior of coaches and team cohesion. Further mediation analysis showed that mental toughness played a partial mediating role between transformational leadership behavior and team cohesion. This finding not only explains the relationship between transformational leadership behavior, team cohesion and mental toughness, enriches the field research of coach behavior, team performance and athletes' psychology, but also provides important guiding significance for sports coaching and training.

The findings of this study provide many insights for the practice of coaches, athletes, and sports managers. First, coaches should be aware of the importance of transformational leadership behaviors and actively apply these behaviors in daily training and competitions. For example, coaches can enhance team cohesion by motivating athletes, providing personalized support, and clarifying team goals. Second, sports organizations can provide coaches with relevant training and development programs to help them improve their transformational leadership capabilities. In addition, coaches and sports organizations should also pay attention to the cultivation of athletes' mental toughness and incorporate it into training plans to further enhance the overall performance of the team.

To make the findings more generalizable, future research could attempt to replicate this study with larger and more diverse samples in different sports and cultural contexts. Moreover, the study only focused on the relationship between mental toughness in transformational leadership and team cohesion, future studies could explore other possible mediating or moderating variables, such as team communication, team culture, and training environment, which may play an important role in the relationship between transformational leadership behavior and team cohesion. Additionally, the research subjects of this study were student athletes, which may be due to the narrow scope of the research subjects, the collected data did not show demographic differences. However, factors such as gender, age, and training experience also play an important role in sports research, future research could also study the differences and connections between these factors by changing the research subjects, such as professional athletes and adolescent athletes.

## Data Availability

The datasets presented in this study can be found in online repositories. The names of the repository/repositories and accession number(s) can be found in the article/Supplementary material.
